# Renal neutrophil gelatinase-associated lipocalin and kidney injury molecule-1 expression in children with acute kidney injury and Henoch-Schönlein purpura nephritis

**DOI:** 10.3892/etm.2014.1595

**Published:** 2014-02-28

**Authors:** YUE DU, LING HOU, JINJIE GUO, TINGTING SUN, XIULI WANG, YUBIN WU

**Affiliations:** Department of Pediatric Nephrology, Shengjing Hospital of China Medical University, Shenyang, Liaoning 110004, P.R. China

**Keywords:** renal neutrophil gelatinase-associated lipocalin, injury molecule-1, acute kidney injury, Henoch-Schönlein purpura nephritis, children

## Abstract

The aim of this study was to investigate the expression of neutrophil gelatinase-associated lipocalin (NGAL) and kidney injury molecule-1 (KIM-1) in the serum, urine and renal tissues of children with acute kidney injury (AKI) and Henoch-Schönlein purpura nephritis (A-on-C). A prospective single-center evaluation of the serum, urine and renal NGAL and KIM-1 levels was performed in a cohort of children. Blood and 5-ml urine samples were collected from each patient for the analysis of NGAL and KIM-1 levels using an ELISA. In addition, the expression of NGAL and KIM-1 in the kidney was examined using immunohistochemistry in patients with A-on-C and HSPN. The expression of serum cystatin C, β2-macroglobulin and serum creatinine (SCr), as well as urinary β2-MG and SCr, in the patients with A-on-C was significantly higher than that of HSPN patients, and the expression of NGAL and KIM-1 in the serum and urine in the A-on-C patients was also significantly higher than that of HSPN patients. However, there were no significant differences in the urine protein levels between the two groups. NGAL and KIM-1 were expressed in renal tubular epithelial cells, and the expression of NGAL and KIM-1 in the A-on-C patients was significantly higher than that in HSPN patients. In addition, the urine NGAL and KIM-1 levels were negatively correlated with glomerular filtration rate, but there was no significant correlation between the urine NGAL/KIM-1 and urine protein levels. The changes in serum and urine NGAL and KIM-1 levels may be applied to the diagnosis of A-on-C.

## Introduction

Acute kidney injury (AKI) is a common critical illness and has demonstrated an increasing trend in its incidence (1). AKI with chronic kidney disease (CKD) is the third reason leading to CKD, following acute tubular necrosis and prerenal AKI (2). The Project to Improve Care in Acute Renal Disease (PICARD) in the United States showed that 30% of patients with AKI were patients with AKI and CKD (3), suggesting that the incidences of AKI and AKI with CKD are increasing annually, as well as increasing in children. Hospital and pediatric intensive care unit (PICU)-acquired prerenal AKI rates appear to have increased >9-fold between 1982 and 2004 (4), most likely due to the increasing use of more invasive management techniques and a higher illness severity of critically ill children. AKI in children is usually caused by renal ischemia, nephrotoxic drugs and sepsis (5). Henoch-Schönlein purpura nephritis (HSPN) is one of the most common types of CKD in children, and may lead to renal insufficiency (6–8). In addition, CKD is one of the factors causing AKI in Chinese children. However, the occurrence of AKI with HSPN (A-on-C) is rarely reported.

Neutrophil gelatinase-assiciated lipocalin (NGAL) is a member of the lipocalin superfamily of proteins that has been extensively studied in AKI. NGAL is one of the most robustly expressed proteins in the kidney following ischemic or nephrotoxic injury in animals (9–13) and humans (14–17). A previous study showed that NGAL was released from tubular cells following various injuring stimuli (18). NGAL has now been validated as an early predictive biomarker of AKI in cardiopulmonary bypass (19), kidney transplantation (16,20), diarrhea-associated hemolytic uremic syndrome (21) and contrast nephropathy (22). However, the expression of NGAL in the serum, urine and renal tissues of children with HSPN and A-on-C, has yet to be elucidated.

Kidney injury molecule-1 (KIM-1) is a sensitive marker of the presence of tubular damage (23). KIM-1 is upregulated in dedifferentiated proximal tubule cells following ischemic or nephrotoxic AKI in animal models, and a proteolytically processed domain may be detected in the urine (24). Tubular KIM-1 expression is significantly associated with tubulointerstitial damage and inflammation (25). In experimental and human kidney disease, increased urinary KIM-1 levels are strongly correlate with tubular KIM-1 expression, showing that urinary KIM-1 levels may be a valuable biomarker for the presence of tubulointertitial damage (26). However, the expression of KIM-1 in the serum, urine and renal tissues of children with HSPN and A-on-C has not yet been investigated.

In the present study, it was hypothesized that the expression of serum and urine NGAL and KIM-1 was significantly increased in patients with A-on-C, with expression in the renal tubules. In addition, it was hypothesized that the urine NGAL and KIM-1 levels negatively correlated with glomerular filtration rate (GFR), although not with urine protein. The study, examined the changes in NGAL and KIM-1 levels in the serum, urine and renal tissues of children with A-on-C, and investigated the correlation between NGAL/KIM-1 and GFR/urine protein levels.

## Materials and methods

### Patients and laboratory data

A prospective single-center evaluation was performed of the serum, urinary and renal NGAL and KIM-1 levels in a cohort of children admitted to the Department of Pediatric Nephrology, Shengjing Hospital of China Medical University (Shenyang, China) between January 2010 and October 2011. Patients were included if they were diagnosed with A-on-C, according to the Pediatric Risk, Injury, Failure, Loss and End-Stage Renal Disease (pRIFLE) criteria (Table I) (27), or with HSPN with nephrotic-range proteinuria, and divided into two groups; group HSPN and group A-on-C. Patients were excluded if they had a known diagnosis of CKD, including dialysis or transplantation, or a recent urinary tract infection. The caregivers of the patients provided informed written consent. The protocol and consent forms were approved by the Shengjing Hospital Institutional Review Board prior to study commencement (no. 20110819).

All relevant data, including age, gender, weight, hemoglobin (Hb), serum creatinine (SCr), cystatin C (CysC), serum β2-macroglobulin (β2-MG), albumin, urinary β2-MG and urine protein levels, were recorded for each of the patients. GFR, also known as estimated creatinine clearance (GFR), was calculated using the original Schwartz formula (28), as opposed to the updated Schwartz formula used in the Chronic Kidney Disease in Children study. This was due to the fact that the original formula was used to validate the pRIFLE AKI classification system (27).

### Blood and urinary biomarker assessment

Blood and 5-ml urine samples were collected from each participating patient for the analysis of NGAL and KIM-1 levels using an ELISA (R&D Systems, Minneapolis, MN, USA), in accordance with the manufacturer’s instructions. The samples were centrifuged at 3,000 rpm for 15 min (Beckman, Brea, CA, USA), and the supernatant was then decanted into 1-ml aliquots and stored at −80°C prior to assay. Urinary Cr was measured using a quantitative colorimetric assay kit (Sigma, St. Louis, MO, USA) to normalize the urine samples.

### Immunohistochemical staining

The NGAL and KIM-1 protein levels in groups HSPN and A-on-C were examined using immunohistochemical staining. The tissue was fixed in 4% formaldehyde and then embedded in paraffin blocks. Slides of kidney tissues measuring 4 μm were routinely prepared for the immunohistochemical analysis of the NGAL and KIM-1 protein levels. A streptavidin-biotin complex (SABC) immunohistochemical assay (Santa Cruz Biotechnology, Inc., Santa Cruz, CA, USA) was performed and rabbit anti-human NGAL antibodies (Santa Cruz Biotechnology, Inc.), which were diluted by a ratio of 1:50, were used as primary antibodies. Based on the SABC staining technique, brownish yellow granules in the cytoplasm indicated positive cells. In addition, an SABC immunohistochemical assay using rabbit anti-human KIM-1 antibodies (Santa Cruz Biotechnology, Inc) as the primary antibodies was performed, with the antibodies diluted by a ratio of 1:160. Brownish yellow granules in cytoplasm indicated positive cells.

Results were analyzed using an image analysis system (MetaMorph^®^ Imaging System; Universal Imaging Corporation, West Chester, PA, USA). As the average integral optical density value increased, the expression intensity of the product with a positive reaction became stronger, indicative of higher protein expression.

### Statistical analysis

Statistical analyses were performed using SPSS 10.0 statistical software (SPSS, Inc., Chicago, IL, USA). All data are presented as the mean ± standard deviation). A t-test was performed for intergroup comparisons. P<0.05 was considered to indicate a statistically significant difference. Pearson or Spearman correlation coefficients were used to assess the correlations between estimated GFR (eGFR) and other variables.

## Results

### Demographics and laboratory data

In total, 25 patients, with an average age of 8.58±2.15 years, were enrolled in the study. Among them, 16 patients had HSPN (group HSPN), while 9 patients had A-on-C (group A-on-C). The renal tissues of 10 patients in group HSPN and 6 patients in group A-on-C were examined using immunohistochemistry. The demographics and laboratory data are shown in Table II. The expression of serum CysC, β2-MG, SCr and urine β2-MG in group A-on-C was significantly higher than that in group HSPN, and the expression of NGAL and KIM-1 in the serum and urine of the patients in group A-on-C was also significantly higher than that in group HSPN.

### Expression of NGAL and KIM-1 in the kidney

**Figure 1:**
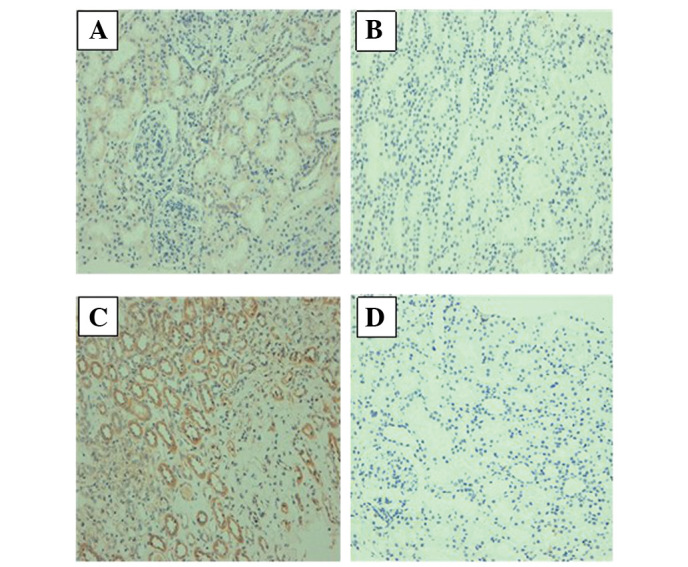
Expression of NGAL in (A) group A-on-C and (B) group HSPN; the expression of KIM-1 in (C) group A-on-C and (D) group HSPN (magnification, ×200). Visualization was performed using DAB chromogen. NGAL, neutrophil gelatinase-associated lipocalin; KIM-1, kidney injury molecule-1; HSPN, Henoch-Schönlein purpura nephritis; group A-on-C, patients with acute kidney injury and HSPN; group HSPN, patients with HSPN.

The NGAL and KIM-1 protein levels were examined using immunohistochemical staining. The results showed that the NGAL and KIM-1 proteins were expressed in renal tubular epithelial cells, and that they were mainly expressed in the proximal tubule, not the distal convoluted tubule or the collecting duct. The expression of NGAL protein in group A-on-C was significantly higher than that in group HSPN. In addition, KIM-1 protein expression was low in group HSPN, while the expression of KIM-1 was positive in group A-on-C (Fig. 1). The results of the quantitative analysis of NGAL and KIM-1 expression are shown in Table III.

### Correlation between urine NGAL/KIM-1 levels and GFR, and NGAL/KIM-1 levels and urine protein

**Figure 2:**
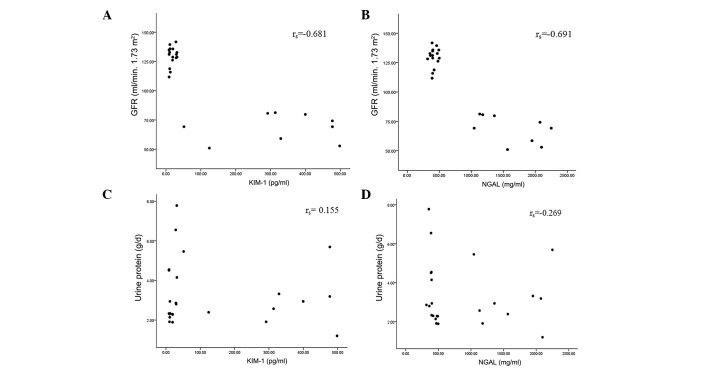
Correlation between urine NGAL/KIM-1 levels and GFR/urine protein. Correlation between (A) urine KIM-1 and GFR (R=0.803), (B) urine NGAL and GFR (R=0.908), (C) urine KIM-1 and urine protein (R=0.083) and (D) urine NGAL and urine protein (R=0.06). NGAL, neutrophil gelatinase-associated lipocalin; KIM-1, kidney injury molecule-1; GFR, glomerular filtration rate.

Pearson or Spearman correlation coefficients were used to assess the correlations between urine NGAL/KIM-1 levels and GFR, and urine NGAL/KIM-1 levels and urine protein. The results showed that urine NGAL and KIM-1 were negatively correlated with GFR; however, there was no significant correlation between urine NGAL/KIM-1 levels and urine protein (Fig. 2).

## Discussion

AKI has become increasingly important in nephrology, particularly in the field of critical kidney diseases. Among adult patients with AKI, patients with AKI and CKD account for ~30% (29). Clinical studies concerning A-on-C in children are rare (30). HSPN is one of the most common types of CKD in children, and may lead to renal insufficiency. However, A-on-C is rarely reported. In the present study, a number of children with HSPN already had AKI when they were admitted to hospital due to infection, hypovolemia or routine urine abnormalities. The differences between the early AKI biomarkers, NGAL and KIM-1, in the serum, urine and kidney of children with HSPN and those with A-on-C have not yet been reported. In addition, there have been no studies concerning the use of NGAL and KIM-1 levels to predict A-on-C, or concerning the correlation between NGAL/KIM-1 levels and GFR/urine protein.

Accurate biomarkers that are able to rapidly detect AKI in children with CKD are likely to be particularly valuable, as baseline SCr values are often unavailable to calculate the required relative increase in SCr to diagnose AKI. In the present study, the expression of serum CysC, β2-MG and SCr and urinary β2-MG and SCr in group A-on-C was significantly higher than that in group HSPN, which was consistent with the expression of NGAL and KIM-1. However, there were no significant differences in urine protein levels between the two groups. A previous study showed that the urine protein levels of children with HSPN was the independent risk factor of prognosis (31). The results of the present study showed that there were no significant differences in urine protein levels between patients with A-on-C and patients with HSPN alone, suggesting that the level of urine protein is not the direct cause of AKI, and may only be used to assess the long-term prognosis. The expression of serum and urine NGAL and KIM-1 in group A-on-C was significantly higher than that in group HSPN, suggesting that NGAL and KIM-1 proteins were highly expressed in patients with A-on-C, and that these proteins may be able to be used as predictive factors for AKI and CKD. The serum CysC and serum and urine β2-MG levels were also increased in patients with A-on-C, and may therefore be combined with NGAL and KIM-1 to predict AKI and CKD. With the pRIFLE formula, it was revealed that while the SCr level was normal in three children, the serum and urine NGAL and KIM-1 levels were significantly increased. This suggested that the increase in serum and urine NGAL and KIM-1 levels occurred prior to the increase in SCr, and may therefore be used to predict AKI and CKD.

From the pathological results, it was demonstrated that NGAL and KIM-1 proteins were expressed in renal tubular epithelial cells, and that the expression of NGAL and KIM-1 proteins in group A-on-C was significantly higher than that in group HSPN. In group HSPN, the NGAL and KIM-1 proteins were not observed in the tubular cells or glomerulus, which contrasted with the high expression in group A-on-C. This indicated that tubular injury had occurred in children with HSPN, which then led to AKI, and that in patients with HSPN alone. The results also showed that NGAL and KIM-1 were primarily expressed in the proximal tubule, not the distal convoluted tubule or the collecting duct, which was consistent with the pathological observations of AKI.

In this study, it was demonstrated that urine NGAL and KIM-1 levels were negatively correlated with GFR, although not with urine protein levels. This suggested that urine NGAL and KIM-1 levels may not be significantly increased in children with HSPN without AKI, even when high levels of urine protein exist. However, in children with A-on-C suffering from infection or hypovolemia, the urine NGAL and KIM-1 levels are likely to be significantly increased. Therefore, the level of urine protein may be associated with the long-term prognosis in patients with HSPN, not the occurrence of AKI. The results suggested that the increase in urine NGAL and KIM-1 levels was associated with AKI, not the CKD or urine protein levels, which was consistent with a previous study (32).

The limitations of the present study were as follows: i) The samples of AKI were small, with no extremely serious cases requiring renal replacement therapy and no classification of AKI; ii) the renal biopsy cases were small, which affected NGAL and KIM-1 protein expression following tubular injury; iii) the study was a single center study, and therefore a further large-scale multi-center clinical study is required to confirm the results.

In conclusion, the expression of serum and urine NGAL and KIM-1 is significantly increased in children with A-on-C. In addition, the results of the present study demonstrated that the levels of serum and urine NGAL and KIM-1 were increased only when AKI occurred in the children, and that NGAL and KIM-1 levels were negatively correlated with GFR. This suggested that changes in NGAL and KIM-1 levels may be used to diagnose A-on-C in children.

## Figures and Tables

**Table I:** pRIFLE AKI criteria.

	GFR	Urine output
Risk ‘R’	Decreased by 25%	<0.5 ml/kg/h for 8 h
Injury ‘I’	Decreased by 50%	<0.5 ml/kg/h for 16 h
Failure ‘F’	Decreased by 75% or <35 ml/min/1.73 m^2^	<0.3 ml/kg/h for 24 h or anuric for 12 h

pRIFLE, Pediatric Risk, Injury, Failure, Loss and End-Stage Renal Disease; AKI, acute kidney injury; GFR, estimated creatinine clearance.

**Table II:** Demographic, clinical and laboratory data of the study population.

Parameter	Group A-on-C	Group HSPN	t-value	P-value
Gender, M/F	3/6	7/9	-	-
Age, years	7.55±1.42	9.50±2.75	1.96	0.062
Weight, kg	28.72±9.28	34.62±13.52	1.16	0.258
Hb, g/l^a^	110.44±6.62	127.94±6.82	6.22	<0.001
SCr, μmol/l^a^	91.22±16.34	47.31±10.92	8.07	<0.001
CysC, mg/l^a^	2.53±0.86	0.80±1.56	7.92	<0.001
Albumin, g/l	32.51±4.93	37.73±3.69	2.77	0.016
Serum β2-MG, mg/l^a^	5.79±3.22	1.63±0.28	5.23	<0.001
GFR, ml/min/1.73 m^2^^a^	68.60±11.78	129.56±8.21	15.23	<0.001
Urine β2-MG, mg/l^a^	14.01±18.92	0.61±0.31	2.88	0.008
Urine protein, g/d	3.18±1.50	3.35±1.73	0.24	0.813
Serum NGAL, ng/ml^a^	312.82±33.17	211.16±34.63	7.15	<0.001
Urine NGAL, ng/ml^a^	1627.61±470.83	412.91±52.36	10.39	<0.001
Serum KIM-1, pg/ml^a^	147.91±68.15	9.28±1.39	8.27	<0.001
Urine KIM-1, pg/ml^a^	329.21±57.36	18.18±9.07	8.02	<0.001

a P<0.05, A-on-C vs. HSPN.

M, male; F, female; Hb, hemoglobin; SCr, serum creatinine; CysC, cystatin C; β2-MG, β2-macroglobulin; GFR, glomerular filtration rate; NGAL, neutrophil gelatinase-associated lipocalin; KIM-1, kidney injury molecule-1; HSPN, Henoch-Schönlein purpura nephritis; group A-on-C, patients with acute kidney injury and HSPN; group HSPN, patients with HSPN.

**Table III:** Renal expression of NGAL and KIM-1 in the two patient groups.

Parameter	Group A-on-C (n=6)	Group HSPN (n=10)	t-value	P-value
NGAL^a^	0.47±0.11	0.03±0.10	13.417	0.00
KIM-1^a^	0.64±0.14	0.03±0.14	13.875	0.00

a P<0.05, A-on-C vs. HSPN.

NGAL, neutrophil gelatinase-associated lipocalin; KIM-1, kidney injury molecule-1; HSPN, Henoch-Schönlein purpura nephritis; group A-on-C, patients with acute kidney injury and HSPN; group HSPN, patients with HSPN.
